# Working With Value Complexity in Healthcare

**DOI:** 10.1177/10497323251330999

**Published:** 2025-04-10

**Authors:** Lieke Oldenhof, Violet Petit-Steeghs, Rik Wehrens, Sander van Haperen, Marjolijn Heerings

**Affiliations:** 1 Erasmus School of Health Policy and Management, 6984 Erasmus University, Rotterdam, The Netherlands; 2 Faculty of Social and Behavioural Sciences, 1234 University of Amsterdam, Amsterdam, The Netherlands

**Keywords:** values, value complexity, new pragmatism, complexity theory, ethnography, secondary analysis

## Abstract

In this paper, we argue for a rethinking of complexity in healthcare in terms of value complexity. Although widely adopted in healthcare, the positivist roots of complexity theory have thus far limited its applicability to understand the wicked nature of many healthcare challenges. We draw on a new pragmatist approach to contend that values are situated and embedded in practice, and demonstrate how values and practices co-develop in ways resembling complex systems. We employ this analytical lens in a secondary analysis of prior ethnographic and action research undertaken between 2010 and 2022 in the context of Dutch healthcare. These studies were conducted in the context of older person care and the (care) services for people with chronic diseases, disabilities, and mental illness. Based on our findings, we bring forward a layered conceptualization of value complexity. Additionally, we illustrate three ways in which practitioners and scholars manage value complexity: “working around,” “working against,” and “working with” complexity. We consider the (dis)advantages of each of these strategies. We conclude that “working with” complexity is difficult in practice yet provides untapped potential to responsibly manage value conflicts.

## Introduction

The healthcare field has increasingly embraced the analytical potential of complexity theory to better understand the multiple challenges that the field currently faces, such as sustaining planetary health, improving pandemic preparedness, and addressing health inequalities. In contrast to simple systems that are highly predictable, complex healthcare systems are characterized by the unpredictable ways in which these systems evolve due to dynamic and emergent interrelations between multiple components (Braithwaite et al., 2018; Greenhalgh & Papoutsi, 2018; Kannampallil et al., 2011; Plsek & Greenhalgh, 2001). Since these interrelations could lead to unexpected outcomes, simple deduction by studying the individual components of a system has been found insufficient (Kannampallil et al., 2011; Plsek & Greenhalgh, 2001). By viewing healthcare as a dynamically evolving ecosystem (Cohn et al., 2013), it becomes possible to move beyond linear “pipeline” models of knowledge implementation and health research (Braithwaite et al., 2018).

Despite increased traction of complexity theory in healthcare over the past 30 years (Papoutsi et al., 2020), theorization and empirical examination of complexity remain suboptimal in health services research (Greenhalgh & Papoutsi, 2018). Stemming from its positivist origins, systems-based thinking tends to reduce complexity by modeling it in mathematical terms (interdependencies between system components), while being unable to fully capture and make sense of social and political dynamics, such as disagreement about the framing of problems and solutions or conflicts between stakeholders. In this light, Tsoukas (2017) and Greenhalgh and Papoutsi (2018) have urged health researchers to further theorize what complexity entails with an open-world ontology that embraces value pluralism and the performative nature of reality.

In this perspective paper, we respond to this recent call by theorizing and empirically unraveling a particular form of complexity that so far has received little attention in applications of complexity theory in healthcare: value complexity. Value complexity, also known as normative complexity, is the existence of multiple, often conflicting values that actors in complex healthcare systems must pragmatically develop responses to in their daily practices (Cribb, 2005; Cribb et al., 2022; Greenhalgh et al., 2023; Wehrens et al. 2023). According to Cribb et al. (2022), this type of complexity arises when healthcare actors have different views on what is important and valuable for quality of care: “it is not just ‘the way things happen’ in healthcare that is complex, but also ‘what matters’- that is, what is, or should be taken as, valuable or successful in healthcare” (Cribb et al., 2022, p. 1). In their study, Cribb et al. (2022) investigate daily conversations in healthcare organizations that reveal how actors differently value what is important in quality of care. While some actors prefer evidence-based modes of quality improvement, other actors argue that experience-based quality improvement is more valuable.

Going beyond the scope of daily conversations about quality in healthcare, the phenomenon of value complexity can be researched more broadly by focusing on different levels of complex healthcare systems and interactions between these levels, for example, by looking at daily work practices of frontline professionals, interactions in cross-sector networks, or the development of evaluation and accountability criteria in healthcare organizations. In a recent plea, Greenhalgh et al. (2023) therefore argue that scholars should research value complexity by looking at value conflicts in healthcare systems and taking into account its implications for decision-making and policymaking such as mistrust between stakeholders. Value conflicts, however, at the same time are constitutive for democratic decision-making and more inclusive knowledge production in multi-stakeholder settings. For value tensions to be productive and foster innovation (Stark, 2009), the development of strategies that productively work with such value complexity is required. However, it is still unclear which strategies actors develop to deal with multiple and sometimes conflicting values and whether these strategies are enabling or hindering collaborative decision-making and shared knowledge production in healthcare.

We take up this recent plea by theoretically and empirically unraveling value complexity as an analytical category. Building on pragmatist value theory, we highlight how values are embedded in daily practices of healthcare actors and how actors develop responses to conflicting values. In prior ethnographic research projects in healthcare governance and management, we found an abundance of value conflicts, diverging norms of good care, policy tensions and paradoxes, and contradictory perspectives on how to sustain collaboration (a.o. Heerings et al., 2022a, 2022b, 2024; Oldenhof & Wehrens, 2018; Oldenhof et al., 2022; Petit-Steeghs et al., 2021, 2023; Schuurmans, Wallenburg, et al., 2021, Schuurmans, van Pijkeren, et al., 2021; Schuurmans et al., 2023; van Haperen et al., 2021; van Muijden et al., 2025). In a secondary analysis of these cases, we adopt the lens of complexity to tease out what we have come to perceive as a central thread across our work: the notion of “value complexity.” We argue that a better theoretical and empirical understanding of the multiplicity of values and how actors deal with value conflicts in daily practices can enrich current discussions about complexity in healthcare.

In the following section, we further develop an open-world ontology of complexity and introduce pragmatist theory to get a better theoretical grip on value complexity from a practice-based view. Correspondingly, we conceptualize value complexity as layered and embedded in situated practices. This conceptualization is illustrated by empirical examples from various research projects in Dutch healthcare. We end with a discussion of strategies that various actors in healthcare practice have developed to “work with” value complexity and reflect on the implications of value complexity for future scholarship inspired by complexity thinking.

## Theorizing Complexity With an Open-World Ontology

In theorizing complexity, we take inspiration from Tsoukas (2017), who has argued for conjunctive theorizing over disjunctive theorizing in order to develop theories that are able to cope with the complexity of the world. Such conjunctive theorizing does not strive for simplifications and reductions but “seeks to make connections between diverse elements of human experience through making those distinctions that will enable the joining up of concepts normally used in a compartmentalized manner” (2017, p. 148). Conjunctive theorizing is characterized by an open-world ontology (viewing flow, flux, and change as fundamental processes of a world that is always in a process of becoming), a performative epistemology (emphasizing that knowing is always action as agents bring the world forward through the act of making distinctions), and a poetic praxeology (which emphasizes the role of practitioners as “nontrivial agents” who are both shaped by and reshape the practices in which they operate).

Importantly, Tsoukas argues against the dualism inherent in many scientific theories that exclude ethical notions from scientific models because of their supposed irreconcilability. Conjunctive theorizing, he argues, “acknowledges the complicated motives of human action and the moral background of action” (2017, p. 148) as practitioners often experience moral uncertainty about “the right thing to do.” Interactions between practitioners in shaping organizational processes are therefore not merely cognitive by nature, but equally affective and embodied, rooted in a value-laden (moral) orientation stemming from their embeddedness in particular (discursive) practices (Tsoukas, 2017).

## A New Pragmatist Approach to Studying Value Complexity in Complex Systems

Scholars outside the realm of complexity theory have made important contributions to understanding value complexity. These scholars move beyond values as stable individual preferences of actors and embrace an understanding of values as contingent, collectively negotiated, and situated in daily practices. Scholars in the field of empirical philosophy (Mol, 2003, 2008; Mol et al., 2010; Pols, 2006a, 2006b; Pols et al., 2017), valuation studies (Fochler et al., 2016; Helgesson & Muniesa, 2013; Kornberger, 2017), and pragmatist sociology (Boltanski & Thévenot, 2006; Jagd, 2011; Lamont & Mizrachi, 2012; Oldenhof et al., 2014, 2022; West & Davis, 2011) have pointed to values as embedded in situated practices, the emergence of values within layered healthcare systems, and to the strategies actors engage in to provide good care amidst value tensions. So far, these insights have not been synthesized to theorize the notion of value complexity. We bring these insights together under the umbrella of “new pragmatism” (Oldenhof et al., 2022; West & Davis, 2011). “New” pragmatism is inspired by “old” pragmatist philosophy (Dewey, 1913, 1939) that emphasizes the need to research values as embedded and produced in everyday practices as a result of continuous deliberation, valuation, and reflective judgment. While building on old pragmatist thinking, “new” pragmatism adds contemporary insights into the question of how multiple values are shaped in current complex systems which are characterized by interactions between multiple stakeholders, network collaboration, and distributed power.

A new pragmatist approach can enhance our understanding of how different actors in healthcare systems experience the multiplicity of values in their daily practices and engage in developing pragmatic responses to value conflicts. Moreover, this pragmatic approach is attuned to analyzing how values are co-constituted by social structures, interactions, and contexts (Dussuage et al., 2015; Oldenhof et al., 2022), embedded in institutional routines and accountability regimes and reconfigured in practices by action, negotiation, and strategizing. In healthcare, this process of co-constituting, embedding, and gradually reconfiguring values is illustrated by the dynamic relations between different layers in the system through which values emerge and the strategies actors adopt in attempts to bring about “good” care amidst value tensions (Beauchamp & Childress, 2013). What actors perceive as “what is good” develops in a dynamic relation with how values are inscribed in meso and macro layers, such as the organizational or institutional contexts. From a new pragmatist perspective, such contexts are infused with values and norms, even in policies where evaluations of good care are presented in factual, objective, or technical terms (Fochler et al., 2016; Helgesson & Muniesa, 2013). Think, for example, of mission statements of care organizations, policies such as quality frameworks, and instruments such as quality indicators and audit procedures in which values related to shared decision-making, personalized care, or positive healthcare are inscribed. Such contexts are understood to frame healthcare professionals, clients, and informal carers’ reflections on and decisions about their practices, consequently shaping these healthcare practices (Fochler et al., 2016; Heerings et al., 2022b).

Despite the embedding of values in institutional structures, actors have agency. They can comply, resist, or change values promoted in organizational and institutional contexts in their evaluations of care in practice (Fochler et al., 2016). Doing so may influence normative contexts such as policies and mission statements. A clear example is the emerging focus on recovery-oriented care in psychiatry, which was induced partly by people with serious mental illness collectively voicing their experiences (Leamy et al., 2011). Recovery-oriented care now commonly appears in both mission statements of organizations providing care for people with serious mental illness as well as in professional guidelines, competency profiles, and outcome measurements (Le Boutillier et al., 2011). In turn, this shapes the perceptions of clients, informal carers and professionals about what is considered good care. Within this dynamic of emerging values, power differences need to be accounted for, for instance, as some actors perspectives and some values are more easily heard and taken seriously than others (Carel & Kidd, 2014). In sum, new pragmatism provides a theoretical perspective enabling analysis of the dynamic emergence of value tensions within different layers of the healthcare system. In this paper, we conceptualize value complexity as an analytical concept and elaborate on the various strategies that actors develop to manage value complexities and deal with value conflicts within healthcare systems.

## Methods

To deepen the concept of value complexity, we engaged in secondary analysis of prior ethnographic research in Dutch healthcare in the period ranging from 2010 to 2022. This included five studies (see Table 1 for an overview of the case studies). These studies focus on different types of care: care for older persons, supported living for people with intellectual disabilities or serious mental illness, psychiatric care, and care for people who fall in the Dutch policy category “misunderstood behavior” (including people with severe mental illness, intellectual disabilities, and dementia). Two of these cases include care across sectors, including both the medical and social domain and also the safety domain in one case.

**Table 1.** Overview of Case Studies.

Case Studies of Value Complexity	Key Questions	Institutional Domains and Networks Involved	Stakeholders Involved in Value Complexity	Data Collection	Further Details
1. Conflicting Values in National Improvement Program for Older Person Care	How do stakeholders differently value what are “good” policy experiments to improve older person care?	Older person care: Both medical and social care domain Cross-sector networks to improve older person care	University Medical Centers, doctors, older person care homes, policy makers, insurers, representative bodies for older persons	53 semi-structured interviews with 63 stakeholders in all groups (professionals, policymakers, older persons) Document analysis and observations of network meetings	Oldenhof & Wehrens (2018); Oldenhof et al. (2022)
2. National evaluation of the action program local initiatives for people with disoriented behavior	How do actors, involved in the care for people with disoriented behavior, tackle this wicked societal issue and what regional developments arise from these actions?	Care for people with disoriented behavior: Both medical and social care domain and the safety domain Cross-sector networks to improve care for people with disoriented behavior	Stakeholders both on a national, regional, and local level. Four regions were studied in more detail. For example, policy makers, ambulance staff, mental healthcare professionals, experts-by-experience, police officers, general practitioners, health insurer staff, social workers, and triage nurses	103 semi-structured interviews with directors, managers, policymakers, and professionals on the work floor, 3 focus groups with managing directors (*n* = 25), 4 patient journeys consisting of interviews with people with disoriented behavior, their family members, and formal care givers (*n* = 20), document analysis of regional and national policy documents and evaluation reports (*n* = 245) and of stories written by people with disoriented behavior (*n* = 37)	Petit-Steeghs et al. (2021, 2023); van Muijden et al. (2025)
3. Regionalization of older person care in the Netherlands	How to facilitate cooperation between stakeholders in multi-level networks?	Older person care: Both medical and social care domain Cross-sector networks to improve older person care	Doctors, older person care homes, policy makers, insurers, representative bodies for older persons	3 years ethnography in 13 regions in the Netherlands, over 1000 hours of observations and 237 semi-structured interviews	Schuurmans, Wallenburg, et al. (2021); Schuurmans, van Pijkeren, et al. (2021); Schuurmans et al. (2023); van Haperen et al. (2021); van Pijkeren et al. (2021); van der Woerd, Schuurmans, et al. (2024); van der Woerd, Wallenburg, et al (2024)
4. Valuations and ethical tensions on good care in supported independent living for people with serious mental illness, intellectual disability, or older persons	Which ethical tensions emerge in the care relationship in supported living and home care services in the context of dominant valuation regimes on good care?	Care for people with serious mental illness, intellectual disabilities, or older persons. Long-term care domain: Supported independent living and homecare services. Within sector networks	Stakeholders on a local level: Clients, experts-by-experience, professionals, family members, managers, and organizational policy makers (ethnographic work within three care teams and document analysis of policies on the national level)	Semi-structured interviews with professionals (*n* = 29); clients (*n* = 33); experts-by-experience (*n* = 8); family carers (*n* = 15); two photovoice workshops with clients (*n* = 7); interviews policy makers and managers (*n* = 7); participant observation in a team providing supported living to people with intellectual disabilities (12 visits, 65 hours); serious mental illness (12 visits, 19 hours); shadowing homecare nurses (10 visits; 60 hours); group consultation on analysis professionals (3 groups; 24 participants in total); clients (3 groups; 15 participants in total); document analysis on policies (Including national quality and accountability policies of stakeholder representative bodies and professional organizations)	Heerings et al. (2022a, 2022b, 2024)
5. Development of prediction models based on machine learning techniques in a Dutch psychiatric hospital department	How are epistemic differences negotiated by data science and psychiatry practitioners in a hospital-based data-driven initiative?	Medical domain: Psychiatry	University medical center: Psychiatrists, nurses, internal and external data scientists	Ethnographic observations (approximately 200 h), including shadowing data scientists; semi-structured interviews (*n* = 19) with data scientists, psychiatrists, nurses, a data engineer, data manager, consultant, hospital manager, and medical researcher; document analysis (>400p.)	Stevens et al. (2020)
		Collaborations with newly established data science team			

Each of the five studies employed ethnographic methods such as interviews, participant observation, focus groups, and document analysis. The specifics of these settings and methods are described in journal articles covering the primary analysis of this data (see Table 1). Cases 2, 3, and 5 were judged by the ethical board of Erasmus Medical Centre as not in need of ethical approval under Dutch law (MEC-2019-0633; MEC-2019-0139; MEC-2017-540); Case 4 was approved by the ethical board of Erasmus Medical Centre approved under Dutch law (MEC-2017-122); Case 1 was not considered for ethical board approval because of its retrospective nature and the fact that the study did not aim to change human behavior. In all five cases, participants gave informed consent for interviews and observations: either verbally or in written form by filling in a consent form.

Secondary analysis of these case studies was done through abductive analysis which is an iterative process—going back and forth between data and theory—to enable qualitative theory development (Tavory & Timmermans, 2014). During the abductive analysis, empirical data form the five cases were analyzed by using value complexity and conflicting values as sensitizing concepts (Greenhalgh et al., 2023), also known as normative complexity (Cribb et al., 2022). In ongoing iterative discussions between the primary researchers of these studies about similarities and differences between these cases, a conceptualization of value complexity was developed that goes beyond its original focus on complex normative issues in quality improvement that can be addressed through dialogue (Cribb et al., 2022). The broadening of value complexity was necessary to explain how (conflicting) values are institutionally and organizationally embedded in different layers of the healthcare system and develop over time through a broad variety of strategies of healthcare actors. Different types of strategies for dealing with value complexity were identified (beyond only dialogue). For peer deliberation, the emerging conceptualization was discussed with other researchers working on similar issues during two workshops, which further refined the analysis, leading to a layered conceptualization of value complexity.

## Value Complexity: A Layered Conceptualization

Based on secondary analysis of these ethnographic studies, we developed a layered conceptualization of value complexity. We start at the most “basic” level, stating that value complexity arises from the existence of diverse values that can be in tension or even in conflict. Each new layer of value complexity subsequently adds an additional “layer” to this basic value complexity. The resulting conceptualization not only describes different forms of value complexity but also shows how value complexity is entangled in practices.

### Emerging Value Conflicts

A first layer of value complexity in healthcare arises from a multiplicity of values, such as safety, personalization, efficiency, patient-centeredness, and empowerment. Moreover, each of these values is subject to different interpretations. What quality of care entails in a particular context often cannot be established unequivocally. Interpretations of the notion of person-centered care in health sciences research, for instance, shift between efficient, self-reliant, participatory, and responsive (Petit-Steeghs, 2019). These differences have an impact on how the patient–professional relationship is constituted and the role that is attributed to the patient within this relationship.

These multiple values—or interpretations of particular values—do not only co-exist but can turn out to be conflictual in practice. This happens whenever values are misaligned, when one (set of) values becomes detrimental to other (sets of) values. The value complexity arising here is that such conflictual values can no longer simply co-exist. Something or someone needs to give way. Daily practices are abundant with examples. For instance, in long-term care, different values co-exist and may be in tension, such as preventing harm and promoting self-determination and self-reliance (Dwarswaard & van de Bovenkamp, 2015). Such values related to autonomy furthermore are imbued with conflicting meanings, including liberal-individual interpretations foregrounding independence and minimal interference, versus relational interpretations celebrating inter-dependence. Different stakeholders involved in the same care practices may prioritize such values differently, resulting in further value tensions. For instance, a professional foregrounds self-determination with regard to personal hygiene and does not interfere with regard to this, while a parent foregrounds health risks and experiences this practice as neglect. Similarly, professionals within the same team may prioritize values differently, thereby judging each other’s work as substandard, resulting in value tensions between team members (Heerings et al., 2022a, 2022b).

Values of healthcare professionals can also conflict with values embedded in other contexts, such as between professionals working in different layers or departments within an organization or at different organizations. For example, in a study of regionalization of older person care, van Haperen et al. (2021) show how tensions result from different understandings of “collaboration” among professionals in different organizations. For some, collaboration is about working together from different specializations, whereas others think of collaboration as unification through mergers. Forced by workforce shortages to cooperate more closely with other facilities in the region, managers of older person care facilities seek opportunities to benefit from working together. Rooted in specific local histories and different religious backgrounds, stakeholders evoke contrasting stories of what it means to be a good partner. Some managers emphasize values such as providence, stewardship, and community cohesion, whereas others emphasize a shared burden and solidarity. These different values lead to conflicting strategies: some pursue mergers and standardization while others seek to specialize and diversify.

A second layer of context giving rise to value conflicts includes the collaboration of healthcare professionals with professionals from other domains. Regarding care provision for people with misunderstood behavior, current policy emphasizes the need for collaborations between professionals from healthcare, social, and safety domains. Within these domains, different values are prioritized. For instance, the safety domain values public order and safety, the social domain livability and self-efficacy of citizens, and the healthcare domain the solicitude and the dignity of individuals (Petit-Steeghs et al., 2021; van Muijden et al., 2025). These different values give rise to different types of measures, hampering the collaboration between domains and subsequently the smooth coordination of services necessary for delivering integrated care (Petit-Steeghs et al., 2021).

Together these examples show the multiplicity of values and their tensions within the healthcare system, which we conceptualize as a first layer of value complexity.

### (In)visibility of Values

A second layer of value complexity follows from the consideration that the visibility of values can vary. In many healthcare settings, different institutional practices—associated with specific values—exist, which are unequally implemented. This results in power differences, as some values are more firmly institutionally embedded, which means that for other values it becomes progressively more difficult to be rendered visible or acted upon. Dominant values in accountability regimes can, for instance, restrain values in professional practice to become visible. An example includes home care services where values such as limiting costs by rationing care are embedded in institutional arrangements and formal instruments. In the time-task model of accounting, care is divided into specific physical caring tasks for which a specific amount of time (in minutes) is available. Such contexts render invisible the work performed by nurses and aides that cannot be accounted for within this model. Examples of this invisible work include the relational work of preventing loneliness by providing social contact and promoting self-determination by developing a relational understanding of older persons’ needs and engaging in dialogue about both life choices and choices related to healthcare. The values put forward in this work such as more relational conceptions of autonomy are trumped by values like efficiency as these later values are well inscribed in formal work procedures such as the time-task model of accounting (Heerings et al., 2022a).

Similarly to professional values, clients’ values can also be rendered invisible within such a power dynamic. An example is in the context of care for people with misunderstood behavior. People with misunderstood behavior assert that diversity in behavior is undervalued in society, expressing underlying values such as tolerance and dignity. These values are less salient in prevailing policies focused on person-centered care, which are instead designed to address “deviance” and promote conformation of behavior to social norms (Petit-Steeghs et al., 2021). Such epistemic injustice with regard to whose values are visible and can be acted upon is very difficult to alleviate (Carel & Kidd, 2014).

Next to power balances, values could also be “hidden” due to the fact that they are entangled with aspects of healthcare practices rendering their normativity invisible. This layer of value complexity thus has to do with the recognition that underneath apparently neutral or objective practices, specific normative aspects can play an implicit role. Normative dimensions play, for instance, an implicit role in professional identity and roles (Berghout et al., 2018, 2020). Implicit ideas about what being a “good” professional entails underly more explicit ideas about methods, expertise, and accepted ways of working within certain professions. An example is described by Petit-Steeghs et al. (2021) regarding the care for people with misunderstood behavior. Professionals mention their struggle with managers’ demands that they should act as entrepreneurs and deliver financial output. These entrepreneurial values clash with professionals’ implicit ideas about their role in delivering good care, having insufficient time to deliver person-centered care. Consequently, professionals conduct activities that are not financially reimbursed in their spare time.

Another way values remain hidden is when they are entangled with epistemic practices. For example, in medicine we witness novel forms of collaboration between medical researchers and data scientists. Following an experiment in which psychiatrists within a Dutch psychiatric hospital department started to work with data scientists in order to develop prediction models of patient outcomes based on machine learning techniques, Stevens et al. (2020) empirically analyzed how differences between epistemic cultures (Knorr-Cetina, 1981) are negotiated in situ. The notion of “epistemic virtues” (Daston & Galison, 2007) served as a sensitizing concept within this research to analyze how discussions between psychiatrists and data scientists were informed by internalized norms (e.g., certainty, representativeness, and objectivity). While respondents would not use the value-laden words like “virtue” or “vice,” these interactions rendered visible how situated judgments were made and to what extent collaboration and mutual understanding became possible (Stevens et al., 2020). This example shows how values are entangled with knowledge practices, for instance, about what is perceived to be normal and acceptable. This means that underneath technical discussions regarding methodological criteria of research, performance measurement systems, or data quality, values about what is conceived to be “proper” research or “evidence” can play a role in the background. Similar implicit value-discussions arising from different epistemic cultures were shown by Petit-Steeghs et al. (2021) between health professionals and patients regarding the development of patient education materials. Where patients valued the intelligibility, completeness, and transparency, health professionals put forward values of correctness, relevance, and uniformity.

In relation to value complexity, these examples make clear how power imbalances impact which values can be rendered visible and how implicit value-judgments can reside underneath explicit ideas within research and health communities about what constitutes “good” knowledge or “good” practice. Variation between this visibility impacts how values can emerge in healthcare systems, thus adding a layer to the conceptualization of value complexity.

### Values Can Change Over Time

A third layer of value complexity relates to the dynamic nature of values over time. This relates to the first and second layer of value complexity since value conflicts and the visibility of values can change over time. In complexity theory, non-linear interactions between system components generate emergent and dynamic properties. Similarly, we suggest that in normatively complex systems, interactions between actors generate emergent and dynamic system properties in the form of values. While values are often presented as relatively stable and durable in legal and ethical scholarship, research in empirical ethics, science and technology studies, and organizational studies has shown that values change frequently, for instance, due to different prioritizations of values in work practices.

An example of how values change over time can be seen in the qualitative analysis of a Dutch experimental improvement program in healthcare (Oldenhof et al., 2022). This longitudinal analysis shows how value conflicts are (temporarily) settled and how different values become more or less dominant in time. For example, while “evidence-based” values were most strongly anchored in the first years of the program, increasing critique and pressure from clients and the funding body allowed for the gradual prioritization of other values, such as empowerment of older persons and inter-organizational collaboration in networks (Oldenhof & Wehrens, 2018).

Another example of changing values is about shifting frames in political debates about people with misunderstood behavior. Initially dominated by incidents that gained prominence in national media with a focus on values of public safety and security, a reframing of the issue called attention to the need for delivering integrated care focusing on values as person-centeredness and carefulness (Petit-Steeghs et al., 2021). Furthermore, their work shows how the perspective of regional stakeholders on political promises of person-centered and integrated care changed over time. Instead of individual responsibility through empowerment and self-realization, values such as public responsibility through collective welfare and camaraderie became more prominent.

Together these examples highlight a temporal dimension to value complexity: values in the healthcare system and their relative visibility or dominance changes over time.

## Dealing With Value Complexities in Practice

Value complexity as conceptualized in this paper thus denotes three layers: first, the multiplicity of values and value tensions are part of the healthcare system and eminent in daily care practices; second, differences in visibility of values; and third, how values and their relative visibility change over time. Since decision-making processes are fragmented in healthcare, managing value conflicts can be highly controversial and contentious in practice. A key question therefore is how various stakeholders in interaction develop strategies to deal with value complexity (Boltanski & Thévenot, 2006; Heerings et al., 2022a; Kornberger, 2017; Oldenhof et al., 2014, 2022). This question can provide valuable insights into the variety and scope of strategies to deal with multiple (and often conflicting) values over time. Moreover, it may provide insight into which strategies facilitate learning in complex healthcare systems where power is increasingly distributed across multi-actor networks and layered settings (van de Bovenkamp et al., 2016).

Drawing on our own empirical work and literature about managing value conflicts across different fields (e.g., public administration, sociology, healthcare management, and STS), we suggest strategies can be broadly categorized as (a) “working around,” (b) “working against,” and (c) “working with” value complexity. These strategies can co-exist and change over time.

The first category of strategies for dealing with value conflict focuses on actors that “work around” and avoid value complexity caused by clashing values, norms, and regulations (Berlinger, 2016; Bozeman et al., 2021; Debono et al., 2013; Wallenburg et al., 2019). As such, workarounds can be considered the proverbial canary in the coalmine: when healthcare systems increase in complexity, workarounds are on the increase as well (Debono et al., 2013; Oldenhof et al., 2022). An often-used example of workarounds in healthcare are “pilots.” Because stakeholders are unable to deal with clashing values in existing healthcare systems (for instance, between affordability of care and quality of care), they temporarily create a niche space where normal rules and regulations do not apply and value conflicts can be avoided (Nair & Howlett, 2016; Oldenhof et al., 2022). Another frequently mentioned example are workarounds that professionals create to ensure safety and continuity of care, for instance, by avoiding workflow disruptions (Debono et al., 2013) or flexible interpretation of protocols (Petit-Steeghs et al., 2021; van Muijden et al., 2025). Although workarounds may provide temporary relief and can be seen as a form of first order learning (learning to work around value clashes), they increase the risk that actors in organizations do not take the opportunity to learn collectively nor ensure accountability as workarounds usually remain “under the radar.”

The second category of strategies for dealing with value conflict focuses on actors that “work against” value complexity by rejecting the multiplicity of values and imposing single value solutions on others (de Graaf et al., 2014; Stewart, 2006; Thacher & Rein, 2004). Examples of these types of strategies are, for example, “bias” (the exclusion of alternative values via the development of one dominant policy paradigm) and “technicization” (Stewart, 2006). Along the same line, “colonization” can be defined as a strategy that denies equality between values by systematically prioritizing one particular value for a longer period of time (Habermas, 1987; Oldenhof et al., 2022). An important consequence of value colonization can be that marginal actors representing alternative values are forced to align with the criteria of the dominant valuation scheme. As a result of colonization, actors resemble or mimic the dominant valuation scheme by adopting the same language and metrics of the dominant group. “Policy silence” highlights another strategy in which the conflictual nature of values is denied or remains hidden. For example, in professional competency profiles, quality frameworks, or indicators, multiple values are described separately without addressing their relational tensions. Consequently, the value complexity that frontline workers have to deal with in their daily practice is not sufficiently acknowledged nor supported (Heerings et al., 2022a).

The final category of strategies for dealing with value conflict consists of strategies that “work with” conflicting values by embracing the multiplicity of values. On the micro level of daily care practices, “tinkering” has been proposed as a way of working with value complexity. Tinkering is a reflexive practice involving attentive experimentation with care practices in which different values are brought together. This is an ongoing process as situations are continuously in-flux and experimental care practices need to be adjusted time and again (Mol et al., 2010). As care is often a collective process of clients, various professionals, and informal caregivers, tinkering is necessarily a collective process (Heerings et al., 2022a). Such processes require infrastructures that facilitate reflection, deliberation, and co-design of care practices (Heerings et al., 2022b). Moreover, affective elements are of key importance in these processes, such as trust and interpersonal dynamics, because these create room for playfulness and creative solutions (Petit-Steeghs et al., 2021) to embrace value conflicts.

When addressing value complexity on the meso or macro level of collaboration between different organizations or policy levels, scholars writing about “wicked problems” argue that value differences should be addressed through collaborative and deliberative strategies in which shared values are constructed through multi-stakeholder dialogue (Denhardt & Denhardt, 2000; Head, 2010; Hisschemöller & Hoppe, 2001). Including stakeholders with diverse perspectives and values in the critical reflection can prevent oversimplification (Hisschemöller & Hoppe, 2001) arising from “working against” strategies. However, organizing inclusive deliberative processes is hard work as power differences are difficult to alleviate in deliberative spaces (Head, 2010). It may not always be possible to articulate shared values and responses to complex problems (Bannink & Trommel, 2019). In these situations, frame reflection and sociological imagination are possible ways to cope with value complexity. Bannink and Trommel (2019) describe frame reflection as a rational debate which helps to reduce misunderstandings resulting from a lack of knowledge on different normative positions. Sociological imagination (Wright Mills, 1959) or speculative ethics (Puig de la Bellacasa, 2017) could be helpful reflexive practices for imagining other ways of looking at an issue, which have been repressed by existing value conflicts (Bannink & Trommel, 2019). When focused on values, such critical reflexive practices can support actors in working with value complexity instead of working around or working against it.

Reflexivity is a key part of ensuring critical frame reflection practices (Thompson & Pascal, 2012). In the case of value complexity, this entails the critical reflection on values. Research of Petit-Steeghs et al. (2021) highlighted the importance of so-called frontier workers in stimulating frame reflection and sociological imagination: professionals who have affinity with different domains or levels in (health)care can make creative connections across those domains and levels and enhance reflexivity.

## Conclusion

In line with Greenhalgh et al. (2023) and Cribb et al. (2022), we argue that value complexity is key to understanding complexity in healthcare systems and beyond. In this paper we have responded to recent calls to further theorize and empirically unravel the notion of value complexity in healthcare systems. Based on a pragmatist inspired approach to studying value complexity in real-life healthcare cases, we differentiate three layers of value complexity: (1) the existence of multiple and conflicting values, (2) the (in)visibility of values, and (3) values changing over time. In previous research, these different layers of value complexity were only addressed implicitly. Stakeholders in healthcare must respond to this value complexity in practice. Being aware of the different layers of value complexity can help in finding suitable strategies to deal with value complexity. Based on our empirical work and literature, various strategies were brought forward. We categorized these strategies into the following groups: (a) “working around” strategies, (b) “working against” strategies, and (c) “working with” strategies. While working around conflicting values can provide quick relief in the hustle and bustle of frontline care delivery, such strategies seem unsustainable in the long run: workarounds lack both accountability and organizational opportunities to learn and increase resilience. Strategies that “work against” complexity reject value multiplicities by imposing a single hegemonic value. This creates legitimacy issues and the marginalization of stakeholders who are “colonized” by a particular value regime. Of the three strategies, “working with” complexity appears to offer the most potential for healthcare professionals to learn from value frictions and offer room for multiple values and perspectives. Rather than implying moral relativism or “anything goes,” inclusive deliberation may increase the accountability of how value conflicts are managed.

Our findings have implications for the potential of healthcare organizations to learn and benefit from value conflicts rather than being paralyzed by value complexity. Healthcare organizations could experiment with strategies of “working with” value tensions. This requires first and open acknowledgement of diverging values. Moreover, actors may need supporting structures or platforms (e.g., reflective spaces) through which they come together and reflect on value tensions (Berlinger, 2016; Oldenhof & Bal, 2023). These may be organized on a team level (Heerings et al., 2022b) or, to align with the layered character of value complexity, between multiple organizational levels and different domains, for example, including directors, middle managers, and professionals at the shop floor; different service providers involved in the same patient trajectories; and insurers, inspectors, policymakers, and actors outside the healthcare domain (Oldenhof & Bal, 2023; Petit-Steeghs et al., 2021). Such processes require time and space, which in healthcare systems marked by scarcity is not a given. One way to enable and sustain “working with” strategies could be to embed these reflective spaces within institutional structures, accountability regimes, and work methods. This would require alternative, more reflexive forms of accountability and governance (e.g., Petit-Steeghs et al., 2021). Reflexive forms of governance focus on how to deal with continuous challenges by including the perspectives and values of actors (Feindt & Weiland, 2018). An emerging example of such type of governance is narrative accountability. Instead of ticking-off criteria, narrative accountability places more emphasis on the values and narratives behind numbers (Ubels, 2015). Although the importance of such reflexive forms of accountability and governance is addressed, insights into how these forms could be embedded are still limited and call for further research.

Healthcare practices and policies are inherently value-laden, fraught with tensions, inconsistencies, and emotions. “Staying with” such aspects of value complexity (cf. Haraway, 2016) might not produce the neatly delineated research findings policy makers crave for but does lead to a much richer set of insights that—ultimately—also comes with more potential to act upon responsibly.
